# Changes in Perceived Stress Following a 10-Week Digital Mindfulness-Based Stress Reduction Program: Retrospective Study

**DOI:** 10.2196/25078

**Published:** 2021-05-25

**Authors:** Aarathi Venkatesan, Holly Krymis, Jenny Scharff, Art Waber

**Affiliations:** 1 Vida Health San Francisco, CA United States

**Keywords:** perceived stress, health coaching, digital mental health intervention, digital therapeutics, mobile phone

## Abstract

**Background:**

As the need for effective scalable interventions for mental health conditions such as depression, anxiety, and stress has grown, the digital delivery of mindfulness-based stress reduction (MBSR) has gained interest as a promising intervention in this domain.

**Objective:**

This study aims to evaluate the changes in perceived stress following a 10-week digital MBSR program that combined an app-based digital program with weekly one-on-one remote sessions with a health coach.

**Methods:**

This study used a retrospective, observational design. A total of 229 participants with moderate-to-high perceived stress scores as assessed by the Perceived Stress Scale (PSS)-10 enrolled in the 10-week Vida Health MBSR program. The program included weekly remote sessions with a certified health coach and digital content based on concepts fundamental to mindfulness practice. The PSS-10 was used to evaluate perceived stress. Of the 229 participants, 131 (57.2%) were considered program completers and provided at least one follow-up PSS-10. A secondary analysis examined the changes in stress scores at 6 months. This analysis was restricted to participants who had been enrolled in the program for at least 6 months (n=121). To account for random and fixed effects, linear mixed effects modeling was used to assess changes in stress scores over time. An intention-to-treat approach was used to evaluate the changes in perceived stress across the entire study cohort, including those who were lost to follow-up. In addition, a reliable change index was computed to evaluate the changes in scores from the baseline.

**Results:**

The findings revealed a significant positive association between program time and stress reduction (B=−0.365; *P*<.001) at 12 weeks. We observed an average reduction in stress scores of 3.17 points (95% CI −3.93 to −2.44) by program week 6 and 4.86 points (95% CI −5.86 to −3.85) by program week 12. Overall, 83.2% (109/131) of participants showed a reduction in stress scores by week 12, with 40.5% (53/131) of participants showing reliable improvement at 12 weeks and 47.8% (56/131) of participants showing a shift to a lower stress level category (ie, moderate-to-low stress). The intention-to-treat analysis revealed a significant, although attenuated, reduction in stress scores at 12 weeks (B=−0.23; *P*<.001). Participants who completed more lessons had an increased likelihood of moving down at least one stress level category (odds ratio 1.512, 95% CI 1.056 to 2.166; *P*=.02). In assessing medium-term outcomes, among participants who had completed at least 6 months in the program, 48.8% (59/121) of members provided a 6-month assessment. We observed a significant reduction in stress scores at 6 months (t58=10.24; *P*<.001), with 61% (36/59) of participants showing reliable improvement.

**Conclusions:**

The findings of this retrospective, observational study suggest that a blended, digital mindfulness-based intervention may support program uptake and meaningful, sustained reduction in stress outcomes.

## Introduction

### Background

It is without question that the demand for mental health services has substantially increased in recent times, given the health, social, and economic ramifications of the COVID-19 pandemic [1,2]. Estimates suggest that nearly 40% of the US population will need treatment during their lifespan for anxiety, depression, or other common mental health conditions [3,4]. Most mental health concerns are psychiatric disorders, including anxiety, depression, and other stress-related conditions, that are treatable and, in some cases, preventable [5-7].

Barriers to seek care that are unique to mental health have been well documented in the literature. Digital mental health interventions (DMHIs) delivered via apps, web-based platforms, or text messaging have shown promise in addressing these concerns. They appear to reduce barriers to access conventional forms of mental health services, including cost, mental health stigma, and accessibility [8-10]. In addition, although DMHIs can be heterogeneous in terms of their approach, area of focus, and method of delivery, they appear to be as effective as traditional forms of in-person treatment interventions [11-13].

More recently, there has been a growing interest in the clinical utility of mindfulness-based interventions (MBIs) in improving mental health, stress management, and well-being. Mindfulness meditation is the act of purposefully paying attention to the present moment and being aware of mental states and processes with a sense of openheartedness, curiosity, and kindness in a nonjudgmental manner [14,15]. It has been proposed that increased awareness and nonjudgmental acceptance of experience facilitate emotional regulation and overall well-being [16,17]. Mindfulness-based stress reduction (MBSR) was originally developed as a treatment intervention for reducing continual stress that accompanies chronic pain [14,18]. However, growing evidence suggests that MBIs are as effective in improving both physical and mental health outcomes [16,19,20].

Research on MBIs and mental health outcomes has spanned two decades, and the general consensus has been that MBIs have a significant effect on improving stress and anxiety [21]. However, research focused on the efficacy of app-based or digitally delivered MBIs is still nascent. Recently, in a randomized controlled trial, Flett et al [22] reported short-term improvements in depressive symptoms among college students assigned to a digital mindfulness intervention compared with a control group. Despite the abundance of mindfulness-based DMHIs, Mani et al [23] noted that part of the challenge in evaluating these programs is that they differ substantially in content, particularly with respect to supporting core mindfulness practices, such as acknowledgment of thoughts and emotions, guided meditations, breath awareness techniques, body scans, and yoga movements. This content variability limits efforts to systematically evaluate the efficacy of mindfulness-based DMHIs. Indeed, a recent study by Cavanagh et al [24] observed improvements in perceived stress, anxiety, and depression among university staff and students who participated in a 2-week self-guided MBI compared with a wait-list control. However, the study found no differences in mental health outcomes between the type of treatment intervention: the intervention arm that incorporated guided mindfulness practice and psychoeducation was no more effective than the psychoeducation-only treatment group. These findings suggest that digital MBIs may differ in their effectiveness from their traditional counterparts because they use different subsets of activities and practices. Despite these inconsistencies, MBIs appear to be effective in stress management and well-being.

In their meta-analytic review of web-based MBIs, Spijkerman et al [25] observed a moderate effect of MBIs on stress (g=0.51), suggesting that digital MBIs can be effective in reducing perceived stress. However, the review also noted that most digital MBI studies include brief interventions, ranging from 2 to 8 sessions, with adherence rates between 35% and 92%. A subgroup analysis observed a larger effect size when comparing provider-supported interventions (g=0.89) with those lacking therapist guidance (g=0.19). These findings suggest that digital interventions that incorporate support by a therapist or coaching may facilitate program efficacy and adherence.

Health coaching has recently emerged as a promising behavioral intervention for improving health outcomes and adherence to mobile app platforms [26,27]. Defined as “a patient-centred process that is based upon behavior change theory and is delivered by health professionals with diverse backgrounds” [26], health coaching is a supportive model in teaching evidence-based interventions, which improve health outcomes by providing individuals with the knowledge, skills, and confidence to manage their health conditions [28-31]. Several frameworks are used by health coaches in their intervention approach, frequently including motivational interviewing and solution-focused goal setting [32-34].

In summary, although there exists an established body of research evaluating the effectiveness of digital MBIs, there has been less focus on the emerging trend of exclusively remote individualized health coaching combined with digital mindfulness tools. Furthermore, there is considerable variability in the structure of digital mindfulness interventions (eg, guided practice vs psychoeducation only and intervention duration). In this study, we evaluated the Vida Health MBSR digital intervention for moderate-to-high perceived stress. Vida Health is a commercially available app that offers tailored digital health programs paired with one-on-one coaching with health education specialists or licensed therapists. The Vida Health app is available in all 50 states with program offerings, including MBSR, cognitive behavioral therapy for depression and anxiety, and chronic disease management.

### Objective

The purpose of this study is to evaluate the effect of a mindfulness-based DMHI program delivered via a smartphone on perceived stress. The program pairs individualized remote health coaching, with tailored lessons and tools to introduce and facilitate the practice of mindfulness. Our primary hypothesis was that participants enrolled in the Vida Health MBSR program would show improvements in perceived stress scores at the end of the program. In addition, we predicted that program engagement, as measured by the extent of coach interaction or program content completed, would be positively associated with reductions in perceived stress scores.

## Methods

### Study Design

This study used an observational, retrospective design to evaluate changes in perceived stress among participants who completed Vida Health app-based and coach-supported MBSR program. Individuals from across the United States were invited to use the Vida platform, paid for by their employers. Participants joined the Vida MBSR program between March 2018 and May 2020. The institutional review board (Western IRB) waived the requirement for informed consent because the study was determined to have minimal risk, and the data were fully anonymized before analysis.

### Outcome Measure

Stress was assessed using the clinically validated 10-item Perceived Stress Scale (PSS-10) [35]. The PSS-10 is an industry-standard assessment instrument designed to measure the perception of stress and how a variety of life situations may occur to them as uncertain, unmanageable, or overburdening. PSS-10 also tracks clients’ feelings and thoughts during the intervening periods between assessments [36].

**Figure 1:**
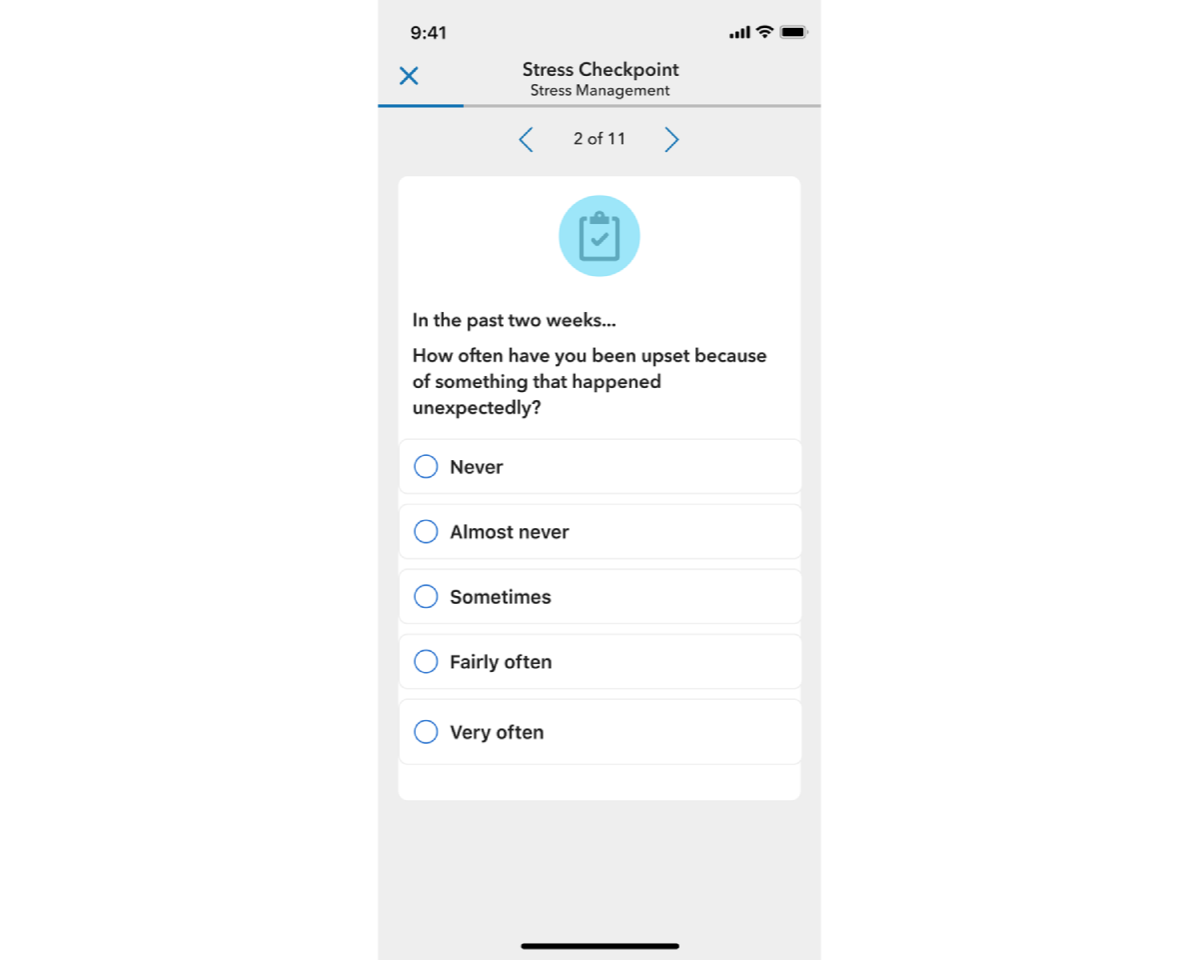
A screenshot of the Perceived Stress Scale-10 assessment in the Vida Health mindfulness-based stress reduction digital program.

The delivery of the PSS assessment occurred via the Vida app (an example of the assessment is shown in Figure 1). The PSS-10 was sent every 2 weeks during the 10-week program intervention and every 3 months in the postintervention phase. Although participants were encouraged to complete the survey on the day on which it was received, they had the option to complete the assessment up to 2 weeks after receipt. After that point, the next scheduled assessment became available in the app.

### Study Population and Recruitment

**Figure 2:**
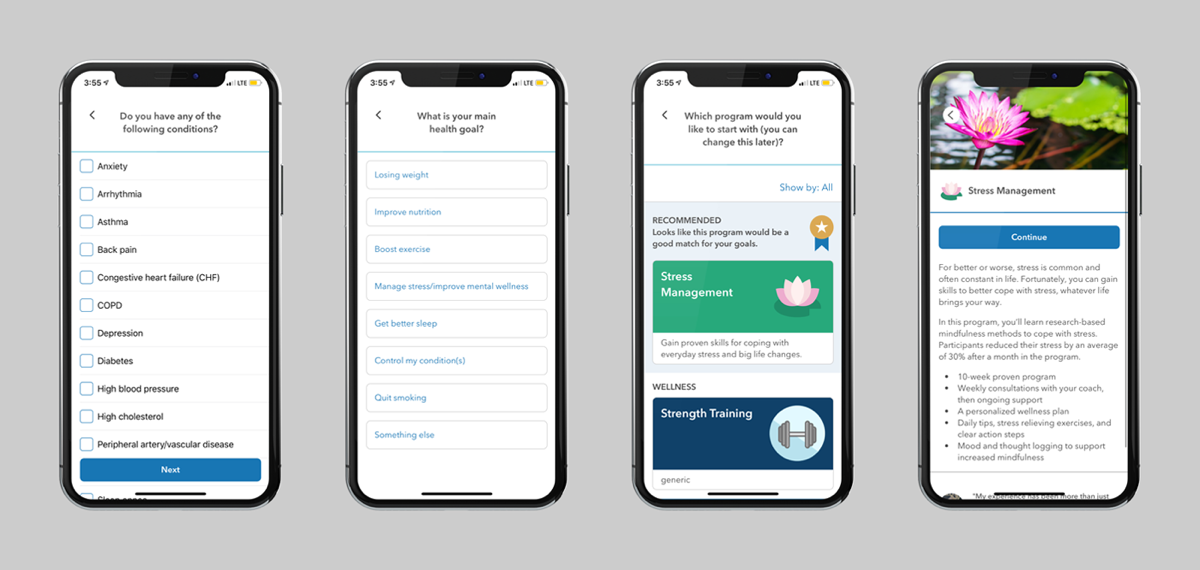
Program screens showing flow into the Vida Health mindfulness-based stress reduction program.

The study included adults aged ≥18 years, who owned a smartphone or tablet, were fluent in English, and had a score indicative of moderate perceived stress or higher at program intake, as measured by the PSS (PSS-10 ≥14). Participants were recruited between March 2018 and May 2020 from employers that offered the Vida Health MBSR program to employees and spouses as part of their health plan. They were recruited through a combination of email announcements, paper flyers, and onsite events at their employer and were directed to download the Vida Health app from the App Store (iOS version) or Google Play Store (Android version). On downloading the app, participants enrolled in the program by completing a brief set of registration questions that included name, contact information, basic demographics (age, gender, height, and weight), existing health conditions, and main health goals (flow shown in Figure 2).

After enrolling in the program, study participants were paired with a certified health coach. Coaches were required to have professional health and wellness coach training from a National Board for Health and Wellness Coaching (NBHWC) accredited program. Coaches performed a video call–based intake in which they determined whether the client was still a good fit for the program. Participants excluded from the study were referred to Vida Health Care Navigators, licensed mental health professionals, who then performed a psychosocial assessment, based on which the participants were referred either internally to the Vida Health Therapy program or external local resources. Conditions that led to exclusion criteria included self-reported moderate-to-severe depressive or anxiety symptoms, reports of suicidality, homicidality or presenting psychosis, active posttraumatic stress disorder, addiction to substances or alcohol, or significant health problems exacerbated by breathing exercises or yoga. Participants scoring in the high perceived stress range (PSS-10 >27) were also offered the option of completing an assessment with a licensed mental health professional to discuss treatment options (remain in the MBSR program, transfer to the Vida cognitive behavioral therapy program for anxiety or connect with external resources to mental health services).

### Study Intervention

Following enrollment, participants received the Vida Health MBSR program, a 10-week digital therapeutic intervention for moderate perceived stress. The intervention was delivered via a mobile app. The evaluated program had three core components: (1) remote personal health coaching with licensed providers, (2) educational lessons and meditation or mindfulness practices, and (3) progress tracking.

**Figure 3:**
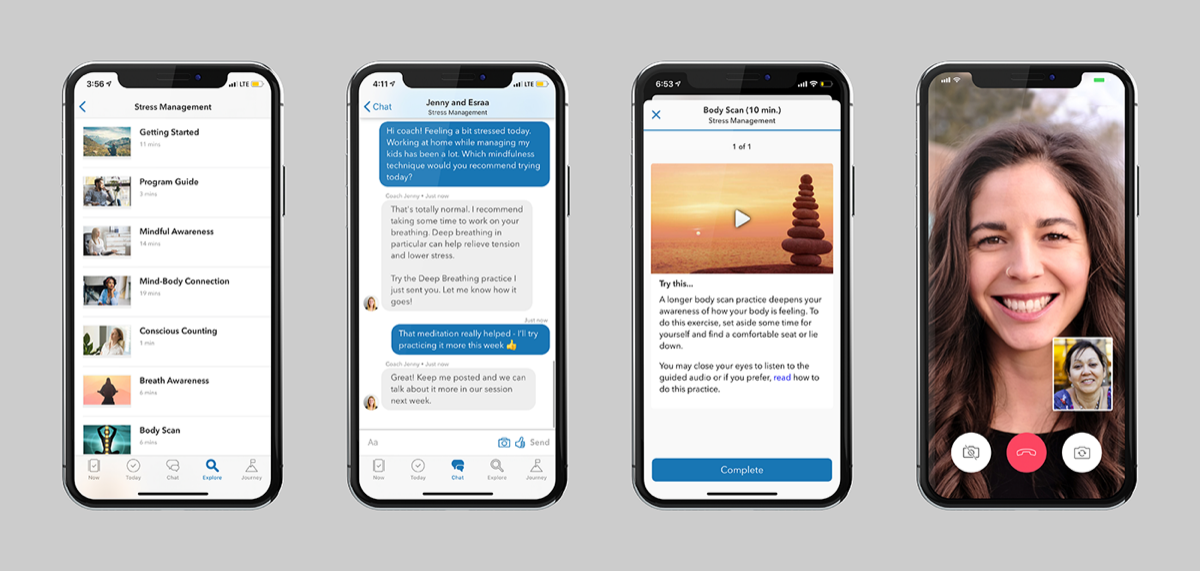
Features of the mobile app available to participants completing the mindfulness-based stress reduction program.

At the start of the MBSR program, participants were asked to select a health coach with whom they would have 30-minute weekly video call sessions throughout the course of the 10-week intervention. During the video call sessions, the coach and participant routinely discussed progress, set goals, and reviewed strategies for applying learned concepts and skills to the participants’ daily lives. In addition to weekly video calls, participants had unlimited app-based messaging support from their coaches. Digital content, which was based on MBSR techniques and could be audio-, video-, or text-based, was automatically sent to participants’ mobile apps every few days. The materials reviewed core concepts of MBSR, including the mind-body connection, meditations, body and breath awareness, and yoga and mindfulness techniques. Health coaches could also send additional content depending on the participants’ needs. Participants were asked to engage with the lessons and practices as they came, but they had access to all of the content throughout the program. Finally, participants were asked to track their stress levels every 2 weeks via the PSS-10 survey. Examples of how the participants experienced these core program components are shown in Figure 3.

The core intervention involved lessons and tools built on concepts fundamental to integrating daily mindfulness-based approaches. These lessons and mindful practices focused on building mindful awareness, establishing healthy relationships with existing stress or stressors, and integrating skills to increase a participant’s autonomy of control over managing and responding to stressors. The lessons and practices were designed for consumption in short intervals, and participants had the option of completing the same lesson or tool multiple times. Were there lessons that were not part of the core?

In addition to the core components of the program, participants received access to various app features to support them in reducing their stress. This included graphs detailing the participant’s progress over time and a home screen that used machine learning models to recommend helpful actions for the participant to take or useful information for the participant to know. These models could prompt the participant to read a specific piece of content, send a message to their coach, log their stress, or view their progress in relation to other members using the Vida app. Participants could also generate habits, or actions to take in real life, that the app reminded them to complete. Participants were able to access and engage with all the functionality of the Vida app during their leisure time.

### Statistical Analyses

All data preparation and analyses were performed using Python version 3.7.9 (Python Software Foundation) and STATA version 16.1 (StataCorp LLC). Baseline was defined as the PSS-10 score completed on the program intake. The week of follow-up assessment completion was computed as the difference in weeks between the survey completion date and the program start date. Baseline differences in stress scores between program completers and noncompleters were assessed using a two-tailed *t* test. As participants were drawn from multiple employers, a Kruskal-Wallis test was performed to assess any potential differences between organizations.

The assessment schedule allowed the participants to complete the PSS-10 survey at any time in a 2-week period. In other words, a participant could complete the survey on the day of receipt or 10 days thereafter. Owing to this inconsistent cadence of completion, only a subset of participants completed the survey in any given week. We used curve fitting, a previously described data imputation technique [37], which can be used to address data sparsity. In brief, we first compiled the time series of PSS-10 scores for each participant. Using the curve_fit function from the SciPy library for Python version 3.7.6 [38], 3 different functional forms (linear, quadratic, and sigmoidal) were fit to each participant’s data. The fit that yielded the lowest root mean squared error was selected for that participant. This procedure was applied to all participants, and the resulting curves yielded data for all weeks that the participant was in the program.

Program engagement was operationalized as two variables: the number of coach consultations and the number of core lessons completed during the intervention. The program engagement factors were each scaled. As described in an earlier paper [39], to adjust for a significant right skew, the number of completed lessons and the number of messages sent were right Winsorized at the 99th percentile. This method retains superusers who may complete many lessons during the program but prevents any one participant from excessively influencing the analyses.

To determine the effect of program time and engagement on changes in perceived stress scores, we used a linear mixed effects model (MLM). Linear MLMs address potential heterogeneity in the data because of possible differences across organizations and provider effectiveness. In this analysis, employer organization and health coaches were entered into the model as random effects. Change in PSS-10 scores from the baseline was entered into the model as the outcome variable. Fixed factors included baseline PSS-10 scores, program time in weeks, gender, number of lessons completed, and number of coach consultations. The reliable change index was computed to estimate the proportion of participants who experienced a reliable improvement in stress outcomes at the end of the intervention and at 3 months post intervention [40]. All analyses were performed using the StatsModels module of Python version 3.7.6 [41] and STATA version 16.1.

## Results

### Overview

**Figure 4:**
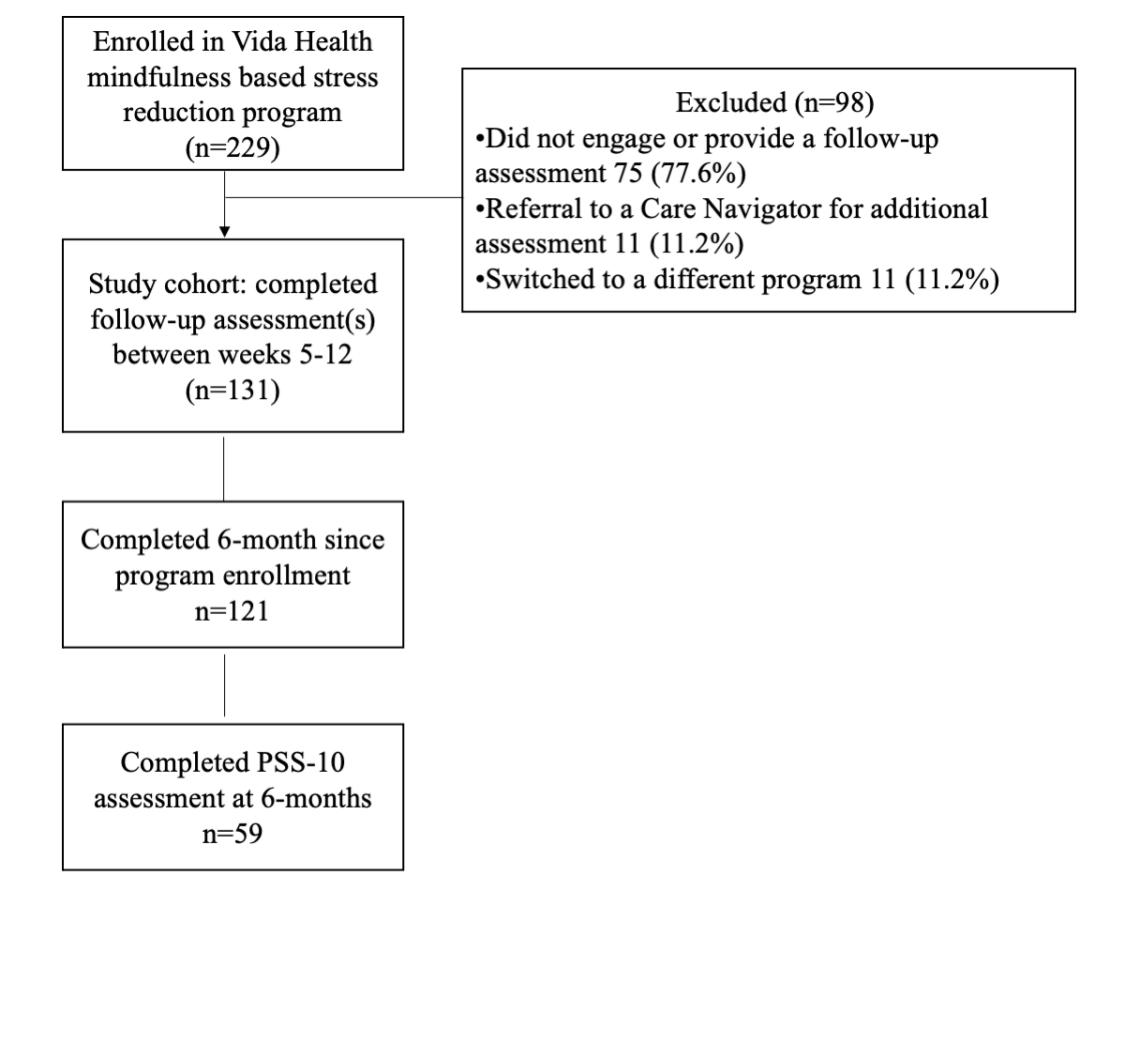
A schematic of participant flow. PSS: Perceived Stress Scale.

A total of 229 participants with a baseline PSS score ≥14 (moderate stress or higher) were enrolled in the Vida MBSR program between March 2018 and May 2020. Study enrollment was restricted to participants who completed at least one in-app program lesson or had at least one coach interaction (video consultation or text message to coach) during the study period. The PSS-10 score reported at program intake was used as the baseline PSS-10 score. A schematic of the participant flow is shown in Figure 4. Of the enrolled participants, 42.7% (98/229) failed to complete at least one follow-up PSS-10 assessment after the first month of the program. In the absence of follow-up assessment, these participants were considered program noncompleters and were excluded from all subsequent analyses. Unless otherwise noted, analysis was restricted to the treatment cohort, defined as participants who completed at least one follow-up PSS assessment within 5 to 12 weeks from the start of the program. Overall, 57.2% (131/229) of the participants met the inclusion criteria in the treatment cohort.

To address potential systematic baseline differences between program noncompleters and the treatment cohort, we performed a two-tailed *t* test that showed a nonsignificant difference in baseline PSS-10 scores between groups (*P*=.10). However, a two-tailed chi-square test suggested that more program noncompleters scored in the higher perceived stress range (PSS-10 ≥27) than participants in the treatment cohort (χ^2^_1_=5.1; *P*=.02). This is expected, as participants scoring in the higher severity range were typically referred to external services or Vida Health Therapy rather than remaining in the MBSR program. There were no significant gender (*P*=.20) or age-based baseline differences between groups (*P*=.45). As noted earlier, participants were drawn from employer-based organizations. The results of the Kruskal-Wallis test showed no significant differences in baseline PSS-10 scores between organizations (*P*=.38).

### Baseline Characteristics

The treatment cohort comprised 131 participants. Baseline characteristics are presented in Table 1. Of 131 participants, 121 (92.4%) reported experiencing moderate stress at baseline, with 10 (7.6%) reporting high perceived stress at baseline. The study included more women than men. There was a marginally significant trend, suggesting that women reported higher levels of perceived stress than men at baseline (t_129_=1.93; *P*=.06). All participants engaged with their coach either via text messages or remote consultations. Of 131 participants, 96 (73.3%) participants had completed at least one consultation with their coach during the intervention period and 130 (99.2%) participants had messaged their coach during the program intervention. A summary of program engagement is presented in Table 2.

**Table 1:** Demographic characteristics of treatment cohort (N=131).

Characteristics		Values
**Gender, n (%)**		
	Female	82 (62.6)
	Male	49 (37.4)
**Age (years), mean (SD)**		
	Female	38.02 (10.92)
	Male	40.75 (10.72)
**Baseline** **Perceived Stress Scale** **-10, mean (SD)**		
	Female	38.02 (10.92)
	Male	40.75 (10.72)

**Table 2:** Summary statistics for program engagement (N=131).

Variables	Consultations (mean 3.87, SD 3.29)		Messages (mean 28.57, SD 5.35)		Lesson completions (mean 10.56, SD 8.21)	
	Correlation coefficients	*P* value	Correlation coefficients	*P* value	Correlation coefficients	*P* value
Consultations	1.0	N/A^a^	0.040	.64	0.094	.28
Messages	0.040	.64	1.0	N/A	0.353	<.001

^a^N/A: not applicable.

### Perceived Stress Outcomes

**Figure 5:**
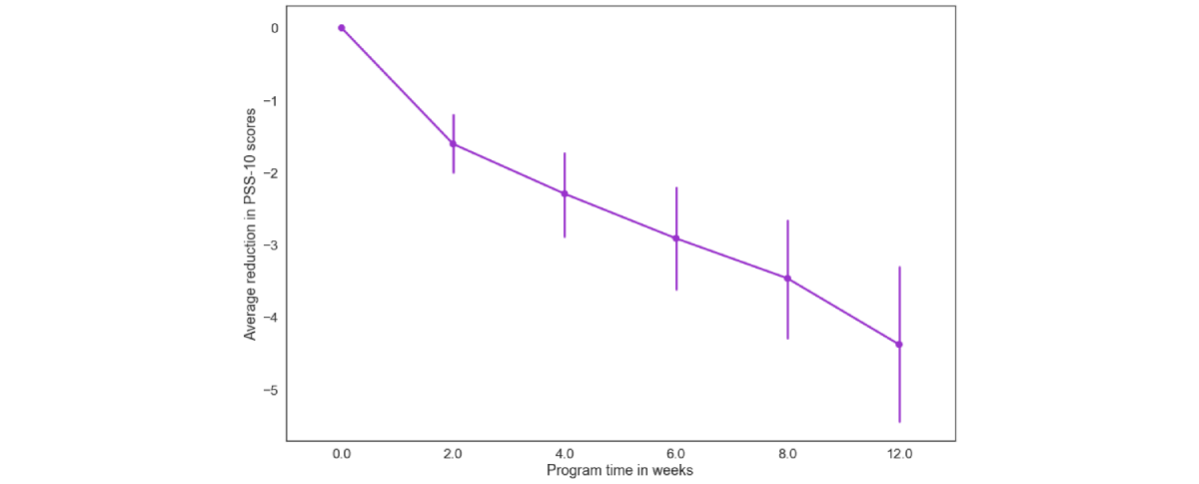
Estimated marginal means of changes in 10-item Perceived Stress Scale as a function of program time. PSS: Perceived Stress Scale.

Overall, 83.2% (109/131) of participants experienced a reduction in PSS-10 scores from baseline by program week 12. Of 131 participants, 56 (47.8%) moved down at least one perceived stress level (ie, moderate-to-low stress) and 53 (40.5%) had a reliable improvement in perceived stress scores. There was a significant effect of program week on reduction in PSS-10 scores relative to baseline (B=−0.365; *P*<.001) such that increased program time was associated with greater perceived stress reduction (Figure 5). We observed an average reduction of 3.17 points (95% CI −3.93 to −2.44) by program week 6 and a reduction of 4.86 points by week 12 (95% CI −5.86 to −3.85). In addition, the analysis revealed a significant inverse association between baseline PSS-10 scores and average reduction (B=−0.402; *P*<.001). Higher baseline scores were associated with greater reductions in PSS-10 scores by program week 12. We observed a nonsignificant trend, suggesting that women showed a greater reduction in perceived stress than men (B=−0.362; *P*=.09).

### Engagement-Based Outcomes

The number of coach consultations had no significant effect on the perceived stress scores (*P*=.69). However, there was a significant effect between core lesson completion and reduction in stress scores by program week 12 (B=−1.420; *P*<.001). Participants who completed a higher number of core lessons had an increased likelihood of achieving at least one-level reduction in perceived stress scores (odds ratio 1.512, 95% CI 1.056-2.166; *P*=.02).

### Medium-Term Perceived Stress Outcomes

In addition, we examined the three-month postintervention outcomes. A total of 92.4% (121/131) of participants had completed at least 6 months from the start of program enrollment. Of this cohort, 48.8% (59/121) provided a follow-up PSS-10 assessment at month 6. A paired *t* test revealed a significant reduction in PSS-10 scores from baseline (t_58_=10.24; *P*<.001). By 6 months, participants showed an average reduction of 6.77 points (95% CI 5.45 to 8.09) in perceived stress scores relative to baseline. A reduction of at least one stress level was observed in 57% (34/59) of participants, and 61% (36/59) of the participants showed reliable improvement in stress outcomes relative to baseline. A Hedges *g* calculation suggested a large effect size (*g*=1.37; 95% CI 0.97 to 1.77).

## Discussion

### Principal Findings

The Vida Health digital MBSR program is an app-based mental health intervention that combines one-on-one weekly remote video sessions with a coach along with tailored digital content based on core concepts of mindfulness practice. The goal of this study is to assess changes in stress outcomes following this MBSR intervention among participants with moderate-to-high perceived stress scores at baseline. The results showed a significant and reliable reduction in perceived stress scores within 12 weeks, which seemed to be maintained at month 6. Higher program engagement, as measured by the completion of core lesson content, was associated with an increased likelihood of a shift to a lower stress-level category (ie, moderate-to-low stress). Although our findings suggest that the Vida Health digital MBSR intervention is associated with improvements in perceived stress, the study design (ie, lack of a control or comparison group) limits our ability to draw causal inferences from these results.

The results of this study are consistent with previous research [12,13,21] that has observed improvements in mental health metrics following digital, app-based interventions. In their meta-analysis, Spijkerman et al [25] observed a stronger effect size for mindfulness interventions that included therapist guidance compared with self-guided interventions. In this study, the frequency of coach consultations was not associated with changes in perceived stress. However, program content completion showed a significant, positive relationship with stress reduction such that participants who completed more core lessons had a greater likelihood of a one-level reduction in stress scores. Coach consults were not correlated with lesson completion; in other words, participants who had more consults did not appear to complete more lessons. We observed a modest, positive association between the number of messages sent to the coach and lessons completed. Although the Vida MBSR program can be self-guided and completed independently of a coach, it is important to note that all participants included in the study had engaged with their coach (either via text messaging or video consultation) during the intervention period, with 73.3% (96/131) having completed at least one consultation. Although not directly evaluated in this study, it is possible that the benefit of coach guidance was in facilitating program uptake and adherence. It has been noted in earlier research that program adherence or retention is a commonly observed challenge of DMHIs, with uptake rates for DMHIs ranging from 0.5% to 28.6% [40]. In this study, we observed that participants who had more coach consultations were more likely to complete postintervention PSS-10 assessments (odds ratio 1.88, 95% CI 1.39 to 2.54; *P*<.001), suggesting that perhaps the coaches serve as a program anchor. In summary, this study observed a significant reduction in perceived stress scores following a 10-week digital MBSR intervention. In addition, the study provides preliminary insight into the role of program content engagement as a possible moderator of this effect. Together, they lend support to the utility and possible efficacy of digital DMHIs that incorporate sound-validated mental health interventions, adding MBSR to the arsenal of options.

### Limitations

This study used a retrospective observational design that lacked a comparison group. The possibility of self-selection bias and the lack of randomization limit the generalizability of our findings and the ability to draw causal inferences regarding the effect of the digital MBSR intervention on stress reduction. As noted earlier, 42.7% (98/229) of the enrolled participants failed to provide an assessment after their initial intake. Although we did not observe any significant systematic demographic differences across program nonstarters and those who remained in the program, it is possible that the groups differed on factors not assessed in this study, such as the presence of other comorbid mental health conditions. We did note that more program nonstarters scored in the high stress range than participants who remained in the program. Although participants with high stress scores were eligible to enroll in this study, participants scoring in the high perceived stress range (PSS-10 >27) were offered the services of a Vida Health Care Navigator to assess the suitability of the MBSR program in addressing their mental health concerns. Indeed, as shown in Figure 4, 22% (22/98) of the program noncompleters had a care navigation consult for external resources to care or switched to a different program. Nevertheless, over three-fourth of program nonstarters failed to engage in the program and provide follow-up assessments. Overall, the observed retention in this study is consistent with previous research that reported an adherence rate ranging from 35% to 92% [25].

Perceived stress was the key outcome metric assessed in this study. However, MBSRs have been associated with improvements in additional measures of mental health, such as depression, anxiety, mood, and well-being [21,22,24]. Additional research is warranted to better define and measure the impact of DMHIs on the treatment of stress management. Future research should incorporate more comprehensive measures of mental health and well-being to better evaluate the possible benefits of digital MBSR interventions. Although we observed a significant positive association between lesson completion and reduction in perceived stress scores, it is possible that participants who experienced improvement were more motivated to complete additional lessons. Moreover, factors not assessed in this study, such as frequency of mindful meditation practice, may account for the observed association between lesson completion and stress scores. In addition, the lessons incorporated psychoeducation and guided practice. It has been suggested that MBSR interventions that incorporate guided lessons may be no more effective than psychoeducation alone [24]. Although research suggests that therapist-supported DMHIs can be as effective as conventional in-person forms of therapy [12,13], further clarity is needed on the role of certified health coaches in MBI programs. Additional research unpacking patterns of engagement in digital interventions, consumption of program content, and their association with mental health outcomes is warranted.

DMHIs through the integration of mindful awareness lessons, practices, and health coaching can be effective in improving mental health care accessibility, cost-effectiveness, and increasing support services to a larger demographic. Future research should involve equivalence trials comparing DMHIs and in-person behavioral health interventions on MBIs for stress management, further examining the importance of the role of health coaches in DMHIs.

### Conclusions

The recent growth and accessibility of smartphones has facilitated the continual development and deployment of mobile-based apps, making it practical for individuals to access the DMHI. Mobile phones facilitate the ability for interventions to enter into the daily lives of individuals, allowing unobtrusive monitoring of activities and contexts, and promote the possibility for interventions at opportune moments, that is, when most needed or desired [42]. Mobile phones are particularly beneficial for mental health care accessibility, as their ownership is largely unrestricted by socioeconomic or demographic status. In addition, they are the preferred form of communication among younger populations, the age group with a decreased likelihood of seeking treatment or support services when affected by mental health conditions [43,44]. MBSR interventions have been shown to be effective in improving mental health outcomes. In this study, adults with moderate-to-high perceived stress completed a 10-week digital MBSR intervention. The intervention paired one-on-one coaching sessions with tailored, guided digital content based on the core concepts of mindfulness practice. We observed significant and reliable postintervention reductions in perceived stress at 12 weeks and 6 months. Although the nonrandomized study design, participant attrition, and the lack of a control group are study limitations, the findings of the study suggest that mindfulness-based digital intervention may be effective in the treatment and management of mental health.

## Abbreviations

DMHI: digital mental health intervention
MBI: mindfulness-based intervention
MBSR: mindfulness-based stress reduction
PSS: Perceived Stress Scale
